# Impact behaviour of freeze-dried and fresh pomelo (*Citrus maxima*) peel: influence of the hydration state

**DOI:** 10.1098/rsos.140322

**Published:** 2015-06-09

**Authors:** Marc Thielen, Thomas Speck, Robin Seidel

**Affiliations:** Plant Biomechanics Group Freiburg, Botanic Garden, Faculty of Biology, University of Freiburg, Schänzlestrasse 1, Freiburg 79104, Germany

**Keywords:** *Citrus maxima*, cellular material, energy dissipation, coefficient of restitution, Maxwell model

## Abstract

Pomelos (*Citrus maxima*) are known for their thick peel which—inter alia—serves as energy dissipator when fruits impact on the ground after being shed. It protects the fruit from splitting open and thus enables the contained seeds to stay germinable and to potentially be dispersed by animal vectors. The main part of the peel consists of a parenchymatous tissue that can be interpreted from a materials point of view as open pored foam whose struts are pressurized and filled with liquid. In order to investigate the influence of the water content on the energy dissipation capacity, drop weight tests were conducted with fresh and with freeze-dried peel samples. Based on the coefficient of restitution it was found that freeze-drying markedly reduces the relative energy dissipation capacity of the peel. Measuring the transmitted force during impact furthermore indicated a transition from a uniform collapse of the foam-like tissue to a progressive collapse due to water extraction. Representing the peel by a Maxwell model illustrates that freeze-drying not only drastically reduces the damping function of the dashpots but also stiffens the springs of the model.

## 1. Introduction

The thick peel of the pomelo (*Citrus maxima*) gives rise to the question of its ‘biological purpose’, especially as it constitutes a large amount of the fruit's volume and thus is quite cost-intensive for the plant in terms of material consumption. Free-fall tests with whole pomelos showed that the peel constitutes—besides other functions—an impact protection layer, preventing the fruit from splitting open when impacting on the ground after being shed, and thereby from being afflicted by mould spores or decaying bacteria [1,2]. This is particularly relevant as on the one hand mould and bacteria reduce the germination capacity of the contained seeds [3] and, on the other hand, destructive fungi and microbes compete with bigger animals for these resources [4,5] and render the fruits unpalatable for potential seed dispersing animals [4,6,7].

From an anatomical point of view the pomelo peel is very similar to that of other *Citrus* fruits [8] with the sole difference that it is much thicker. Apart from the epidermis which—together with few hypodermal cell layers—emerges from the exocarp, it consists of flavedo and albedo, which both emerge from the mesocarp. Whereas the flavedo consists of tightly packed parenchymatic cells, the albedo exhibits considerable air-filled intercellular spaces, gradually changing in size towards the fruit pulp [9]. Even though the albedo exhibits large intercellular spaces, neighbouring cells are in direct plasmodesmatal connection [8]. The albedo also constitutes the largest part of the peel. Except for the lignified vascular bundles running through the whole peel, and the collenchymatous hypodermis, the peel consists of parenchymatous cells which only have a primary cell wall. The cell wall can be regarded as a fibre matrix compound consisting of cellulose fibres and the cell wall matrix, whose main constituents are pectins and hemicelluloses [10]. This cell wall encloses the living tonoplast which is filled with liquid and organelles. Extensive work about the hierarchically structured plant cell walls is available [11–13] and the mechanical properties of cell walls are reviewed in Agoda-Tandjawa *et al.* [14].

Unlike many other biological cellular materials, e.g. apple cortex, which can be regarded as a pressurized fluid-filled closed cell foam [10], cork, which can be regarded as an air-filled honeycomb [15], or trabecular bone, which can be seen as an open celled foam [16], the albedo represents an open celled foam whose struts (consisting of elongated parenchyma cells) are pressurized and filled with fluid. The mechanical properties of the peel are influenced on the one hand by the mesoscopic structure of the tissue but, on the other hand, they also depend on the individual strut cells that become more flaccid owing to displacement of cell fluids out of the cell [17].

Relaxation tests showed that this peel is highly viscoelastic and that its mechanical behaviour can be represented in good approximation by a Maxwell model [18]. This means that the mechanical behaviour can be modelled by means of hookean springs and dashpots. In the specific case the mechanical behaviour of the pomelo peel is represented as a parallel array of one spring and three Maxwell elements, which for their part consist of a spring and a dashpot in series [18].

The aim of this work is to analyse the influence that the water content of the cells (and cell walls), constituting the struts of the foam-like tissue, has on the energy dissipation capacity of the pomelo peel under dynamic loading situations. For this purpose, fresh and freeze-dried peel samples were compared by submitting them to drop weight tests allowing for the calculation of the coefficient of restitution (COR), which is the ratio of rebound velocity to impact velocity of the impactor. This parameter is a constant that depends not only on the material properties of the impacting bodies but also on the impact velocity. It indicates the degree of elasticity of an impact and can take values between 0 (perfectly plastic impact) to 1 (perfectly elastic impact) [19].

## 2. Material and methods

### 2.1. Sample preparation

Cylindrical peel samples used for the drop weight tests were prepared from whole pomelos (*C. maxima* variety ‘honey pomelo’) with the aid of a cork borer (for details see [18]). In order to remove all remnants of the fruit pulp they were trimmed with a razor blade (see also [18]). From each individual fruit, samples for being tested in a fresh state as well as samples for being freeze-dried before testing were prepared, so as to minimize the influence of natural variability of individual fruits on the test results.

### 2.2. Freeze-drying of the samples

Samples were quenched in liquid nitrogen and subsequently vacuum freeze-dried overnight in a Christ Alpha 2–4 LCS freeze-dryer (Martin Christ Gefriertrocknungsanlagen GmbH, Osterode am Harz, Germany) at 2°C and 1.25 mbar. After removing from the freeze-dryer the samples were stored for a maximum of 2 days over silica gel (Carl Roth GmbH+*Co*. KG, Karlsruhe, Germany) until being tested.

### 2.3. Mechanical tests

**Figure 1.**
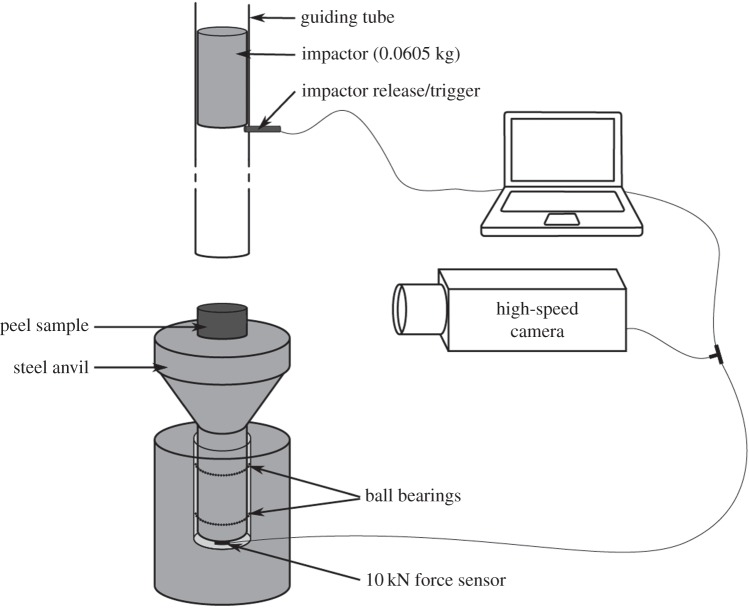
Schematic drawing of the drop weight test rig. The drop height of the impactor is adjustable in order to achieve different impact velocities. The whole test rig was mounted on a massive granite pedestal (*ca* 95 kg).

The dynamic tests were performed on a custom-made drop weight test rig as illustrated in figure 1. It consists of flat-ended rigid aluminium impactor (0.0605 kg) that is dropped from a given height onto the sample. The impact velocity is regulated by adjusting the drop height of the impactor by means of a release pin inserted into holes at specific heights of the guiding tube (0.125 m–1.250 m). The sample is placed on a steel anvil under which a 20 kN force sensor (model 8402–6020, Burster Präzisionsmesstechnik GmbH & Co KG, Gernsbach, Germany) is mounted which records the force exerted by the impactor and transmitted through the sample (raw data in the electronic supplementary material). The sampling rate is 10 kHz. A high-speed camera (model MotionPro Y4, Integrated Design Tools, Inc., Tallahassee, FL, USA) records the impactor striking the sample, at 16 300 fps (four exemplary compressed videos in the electronic supplementary material). The whole system is mounted on a massive granite pedestal (*ca* 95 kg). Releasing of the impactor triggers the data acquisition of the force sensor and the recording of the high-speed camera.

### 2.4. Data evaluation

**Figure 2.**
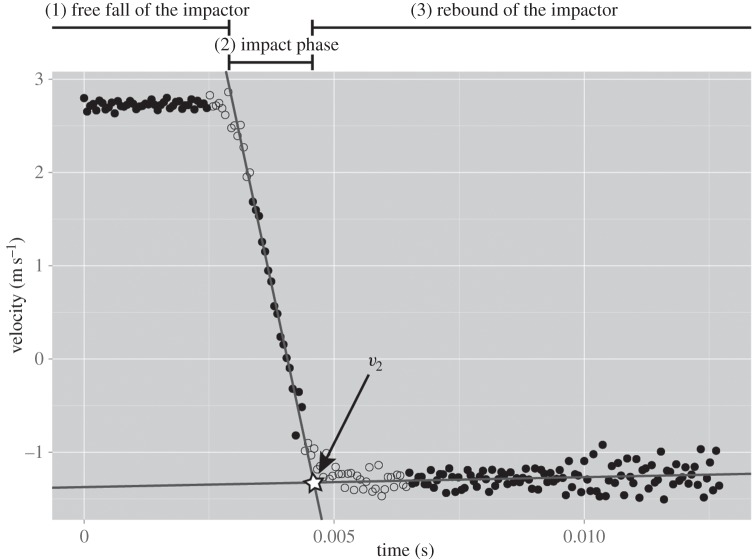
Representative velocity versus time curve of the impactor, covering the three phases of a drop weight test: (1) free fall of the impactor, (2) impact phase and (3) rebound of the impactor. The intersection of the linear regression lines, calculated from the velocity versus time data points of the impact phase and the rebound phase was defined as the rebound velocity (*v*_2_). (The unfilled circles were not used for calculating the regression lines).

The velocity of the impactor (*v*_1_) immediately prior to striking the pomelo peel sample was calculated as follows:

2.1 $$v_{1}=\sqrt{2\;gh},$$

with *h* being the drop height of the impactor and *g* the gravitational acceleration (=9.81 m s^−2^). The rebound velocity (*v*_2_) of the impactor was assessed by analysing the high-speed videos using the motion tracking software Motion Studio 2.11.00 (Integrated Design Tools, Inc., Tallahassee, FL, USA) (raw data in the electronic supplementary material). In order to enable the software to properly track the impactor, the latter was marked with a stochastic pattern. After calibration of the system, using a video frame that shows a reference scale made with exactly the same setting, seven marking points were randomly chosen to be tracked. The velocity of the impactor was calculated by averaging the velocities of the individual track points (figure 2). The intersection of the linear regression lines (the regression can be assumed to be linear as the time period is very short), calculated from the velocity versus time data points of the impact phase and the rebound phase, was defined as the rebound velocity (*v*_2_).

The COR is the absolute value of the ratio of *v*_2_ to *v*_1_:

2.2 $$\mathrm{COR}=\left|\frac{v_{2}}{v_{1}}\right|.$$

The relative kinetic energy (Δ*E*_kin,rel._) that was dissipated by the peel sample during impact was calculated as follows:

2.3 $$\Delta E_{\mathrm{kin},\mathrm{rel}.}=\frac{1/2mv_{1}^{2}-1/2mv_{2}^{2}}{1/2mv_{1}^{2}}=1-{\mathrm{COR}}^{2},$$

with *m* being the mass of the impactor and its kinetic energy *E*_kin_ being

2.4 $$E_{\mathrm{kin}}=\frac{1}{2}mv^{2}.$$

The raw data from the force sensor were processed using the R software package (v. 2.15.3). For each individual drop test dataset, the measured forces were corrected by the baseline offset which was computed from acquired data points before the actual impact (free-fall phase of the impactor, figure 2). The time of the beginning (*t*_1_) and end (*t*_2_) of the impulse was determined manually for each set of data. The impulse (*I*), i.e. the area under the force (*F*) versus time (*t*) curve, is given by integral (2.5) which was solved numerically by applying Simpson's rule.

2.5 $$I=\int_{t_{1}}^{t_{2}}F(t)dt.$$

The accuracy of the force signal was validated by comparing the values of *I* calculated according to equation (2.5) with values for *I* that were calculated by performing a momentum balance (equation (2.6)) [20] (hereafter referred to as *I*_*i*_ and *I*_mb_, respectively).

2.6 $$v_{1}m=I_{\mathrm{mb}}+v_{2}m,$$

Combining (2.5) and (2.6) gives (2.7).

2.7 $$\int_{t_{1}}^{t_{2}}F(t)dt=m(v_{1}-v_{2}).$$

Note that impact velocity (*v*_1_) and rebound velocity (*v*_2_) are vector values and therefore opposed in sign, *v*_2_ being negative.

The maximal compression (i.e. maximum strain) of the peel sample was determined by direct measurements from the high-speed videos using the Fiji image processing package [21]. As high-speed videography faces the trade-off between temporal and spatial resolution, the video frames had a maximum spatial resolution of 1016×260 px. For this reason, the aforementioned measurements were validated by calculating the (maximum) strain indirectly from the force sensor signal using the formula (2.8), which gives the peel sample displacement *x*(*t*),

2.8 $$x(t)=\int_{t_{1}}^{t_{2}}\left(v_{1}-\int_{t_{1}}^{t_{2}}\frac{F(t)}{m}dt\right)\;dt,$$

and

2.9 $$\varepsilon (t)=\frac{x(t)}{l_{0}},$$

which gives the strain *ε*(*t*), with *l*_0_ being the sample height [20].

## 3. Results

**Figure 3.**
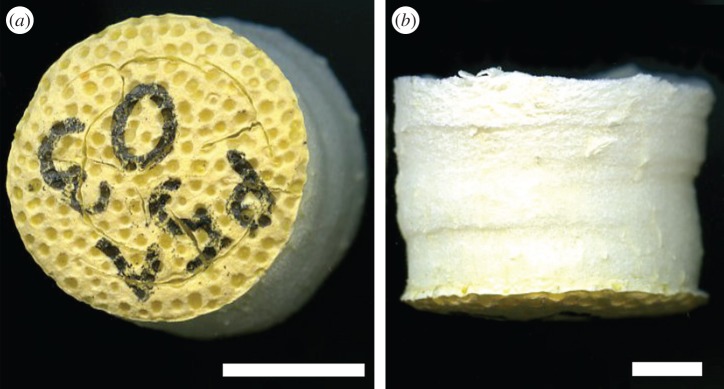
Freeze-dried pomelo peel sample. Top view showing the epidermis where some freeze-cracks are visible (*a*) and lateral view with the epidermis at the bottom (*b*). Scale bars, 1 cm.

Freezing of the peel samples via liquid nitrogen led to freeze-cracking of the peel tissue in some cases (figure 3). Especially the denser regions of the samples, i.e. epidermis and flavedo of the peel, were affected (figure 3*a*). Specimens with visible cracks in the mesocarp were discarded. The mean mass loss owing to water sublimation during freeze-drying was 78.99±1.89%. A tendency was found that higher samples, resulting from a thicker part of the pomelo peel, had a slightly greater weight loss. The reduction in height of the samples was found to be 4.52±5.35%. As the lateral shrinkage was anisotropic (figure 3*b*), it was measured at three different positions: at the epidermis (lateral shrinkage: 10.47±2.29%), at the middle of the sample height (lateral shrinkage: 13.11±2.64%) and at the top of the sample, corresponding to the most inner part of the peel (lateral shrinkage: 10.02±3.01%).

**Figure 4.**
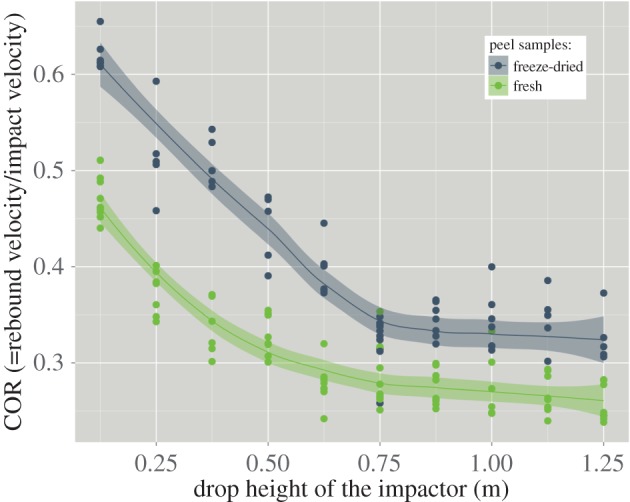
Coefficient of restitution for fresh and for freeze-dried pomelo peel samples versus drop height of the impactor (the lines show the regression of the locally weighted scatter plot smoothing, the light coloured area represents the 68% confidence interval).

The COR is a measure of the elasticity of an impact, and as such it is an important parameter for the analysis of the experiments depicted above. Figure 4 shows the COR for fresh and for freeze-dried pomelo peel samples that were compressed by a rigid impactor that was dropped from different heights. Two facts become obvious: (i) the COR is strongly related to the drop height of the impactor, it decreases with increasing drop height, and thus also with increasing impact velocity, and (ii) freeze-dried samples behave more elastic during an impact than fresh samples.

**Figure 5.**
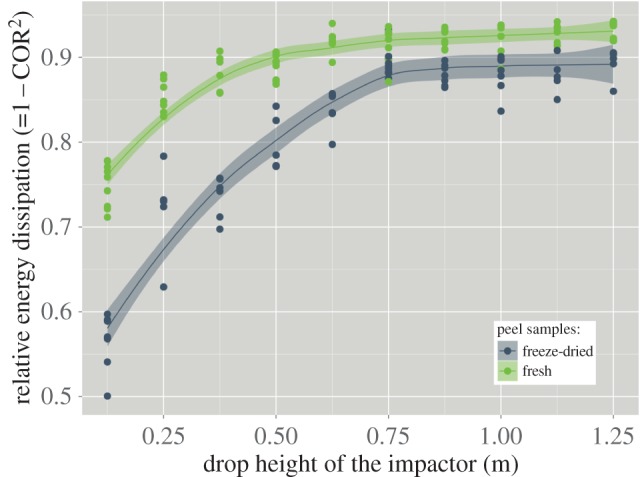
Fraction of the kinetic energy dissipated by the samples during impact against drop height of the impactor (the lines show the regression of the locally weighted scatter plot smoothing, the light coloured area represents the 68% confidence interval).

The relative fraction of dissipated kinetic energy during the impact by the samples is shown in figure 5. For the range of impactor drop heights used during these tests, fresh samples dissipated a greater fraction of the kinetic energy of the impactor (cf. equation (2.3)) than did the freeze-dried samples. For both types of samples the energy dissipation increased with the impactor drop height.

**Figure 6.**
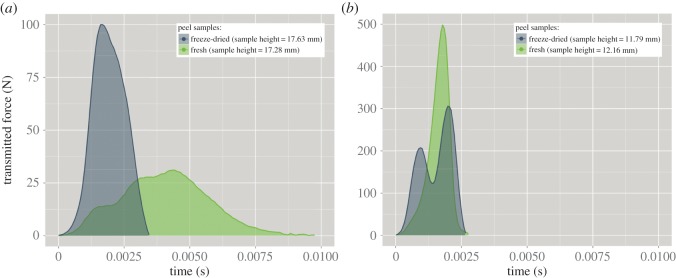
Force transmitted from the impactor through a pomelo peel sample to the force sensor versus time. The impactor was dropped from 0.125 m (*a*) and 1.25 m (*b*). The shaded areas under the curves represent the impulse.

**Figure 7.**
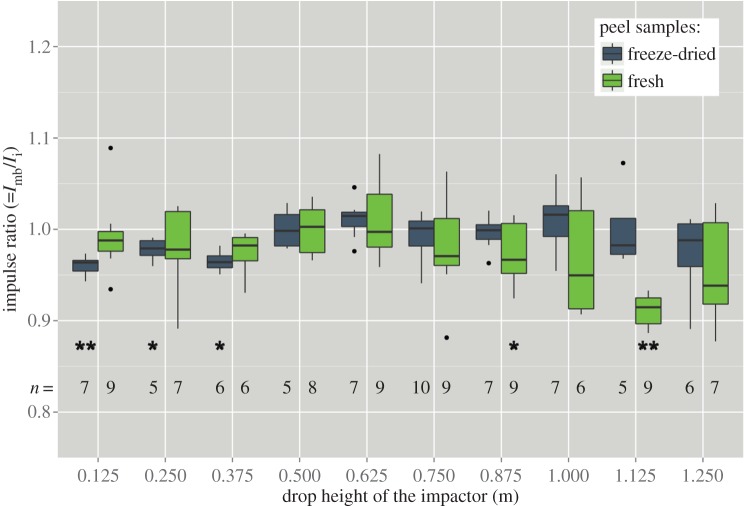
For validation of the force signal via momentum comparison, the momentum as calculated from the momentum balance (*I*_mb_) and the momentum as calculated as the integral of the force versus time curve (*I*_i_) are compared. Ideally, the ratio of both should be exactly 1. Significant differences (Wilcoxon test) from unity are marked with asterisks (*p<0.05, ^**^p<0.01).

**Figure 8.**
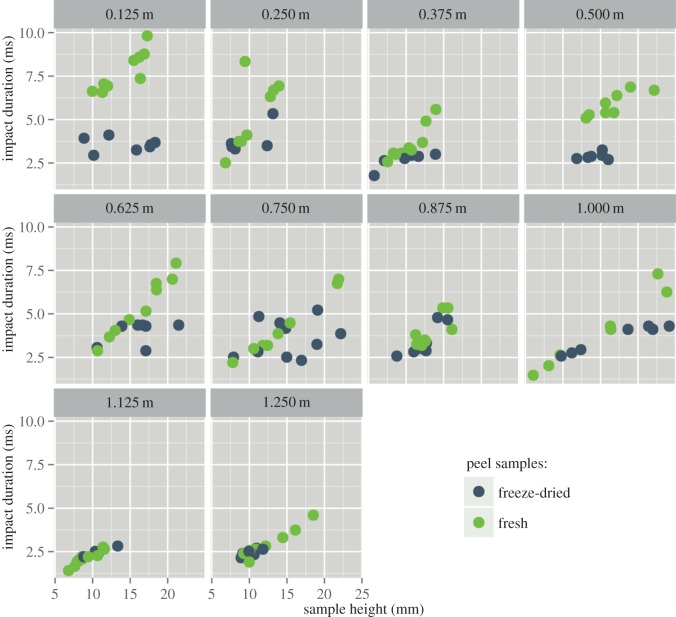
Impact duration versus sample height. Each box represents a different impactor drop height. Each circle represents an individual drop test.

**Figure 9.**
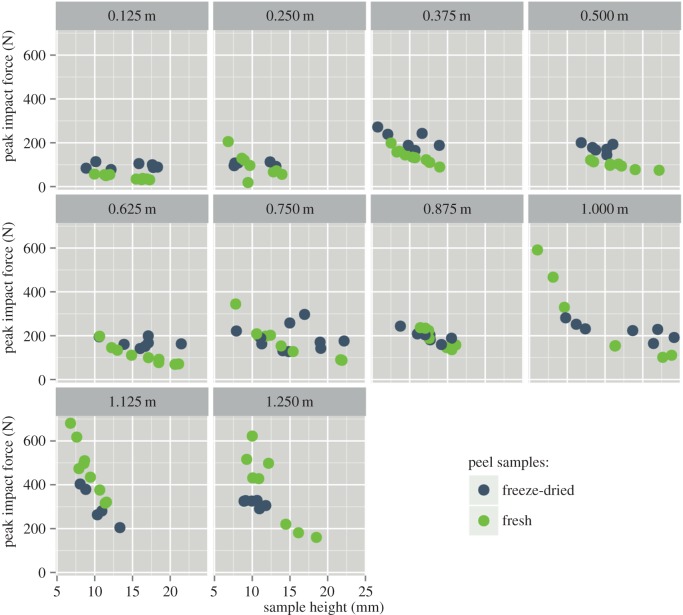
Measured peak force versus sample height. Each box represents a different impactor drop height. Each circle stands for an individual drop test.

Figure 6 shows four representative force versus time curves of the force exerted by the impactor on the pomelo peel samples and recorded by the sensor. The curves originate from tests where the impactor was dropped from 0.125 m (minimal drop height) and 1.250 m (maximal drop height), respectively, onto fresh and freeze-dried samples. For reasons of better comparability, samples with a similar height were chosen for the respective exemplary force versus time curves. In the case of the 0.125 m drop height for the fresh peel sample, the impact duration is longer and the peak force is lower as compared with the same variables in the case of the freeze-dried sample. In the case of the 1.250 m drop height, the fresh peel sample shows a higher peak force, whereas the impact duration is almost the same as for the freeze-dried sample (a detailed presentation of these values can be seen in figures 8 and 9 and will be analysed later). Freeze-drying also led to a double peak in most of the force signals (for the drop heights 0.125 m and 0.250 m, however, it was less pronounced), while fresh peel samples did not entail this feature, except for very few datasets. The area under the force curve (shaded areas in figure 6) represents the impulse and, as can be seen from equation (2.7), is an appropriate method to validate the force signal. If the impulse as calculated from the momentum balance (equation (2.6)) is the same as the impulse as calculated by integrating the force curve (equation (2.5)), which are independent measuring methods, the force signal can be regarded as reliable. The ratio of both values is represented in figure 7.

The individual impact durations of the impactor striking the peel samples are shown in figure 8. It can be seen that for low drop heights, the impact duration is longer for fresh samples compared with freeze-dried samples. In fresh peel samples the impact duration markedly increases with increasing sample height. This tendency is much less pronounced for freeze-dried samples.

Contrary to the impact duration, the peak of the transmitted force tends to decrease with increasing sample height and to increase with increasing impactor drop height (figure 9). Also in this case the relationship is more pronounced in fresh samples. Owing to lateral shrinkage of the samples during freeze-drying, the differences of peak stresses between fresh samples and freeze-dried samples are higher than could be expected from the force versus time curves alone. Stresses in freeze-dried samples would be relatively higher than in fresh samples. As, however, the lateral shrinkage was anisotropic in the tested samples, we forbear from presenting stress calculations for the tested samples.

**Figure 10.**
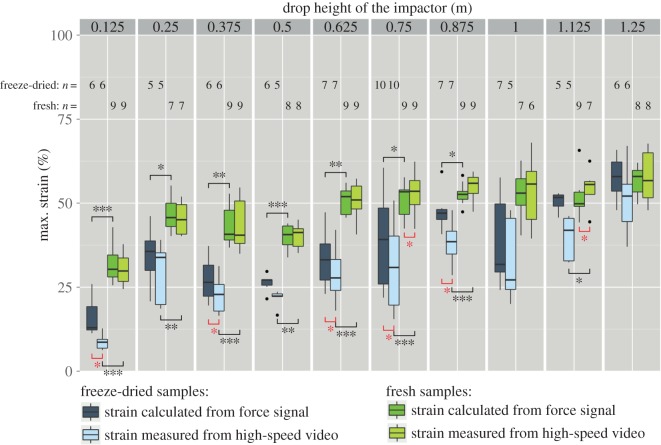
Maximum strain up to which the pomelo peel samples were compacted during impact. In order to compare the different values (difference between measuring method (red asterisks) and difference between sample hydration state (black asterisks)), the Wilcoxon signed-rank test was performed (paired in case of comparison of the different methods). (*p*-values: *p<0.05, ^**^p<0.01, ^***^p<0.001).

Measurement of the maximum strain (e.g. maximum compression) was performed in two different ways: (i) by integrating the force signal (see equations (2.8) and (2.9)) and (ii) by analysing the high-speed videos of the impact. Figure 10 shows that for fresh samples both methods yielded very similar values, with significant differences only at impactor drop heights of 1.125 m and 0.75 m. In the case of freeze-dried samples, the values for maximum strain resulting from the different measuring methods differ more strongly from each other. The differences between fresh samples and freeze-dried samples are significant for drop heights from 0.125 m to 0.875 m, independently from the measuring method.

## 4. Discussion

Freeze-drying of biological (plant) tissues not only removes the water from the protoplast by ice sublimation but also the amorphous cell wall matrix is dehydrated, inducing glass transition, which renders the formerly ‘rubbery’ and viscoelastic cell wall ‘glassy’ and more elastic. Water thus has a severe impact on the mechanical properties of the peel parenchyma, which are mainly governed by the cell wall properties and the turgor pressure [22,23]. The latter results from the protoplast, which is filled with an incompressible and highly viscous fluid, the cell sap, exerting hydrostatic pressure against the cell wall. The turgor loss of the individual cells, as well as contraction stresses resulting from mechanical disequilibrium due to ice sublimation, explain the shrinkage of samples that could be observed. As the parenchymatous tissue of the peel samples exhibits a density gradient [9], the shrinkage was slightly anisotropic (figure 3). During slow freezing ice crystals tend to grow outside cells, entailing damage by cell collapse and rupture [24]. Therefore, and because of the relatively large volume of some samples, we decided to induce fast freezing by quenching the samples into liquid nitrogen, which resulted in freeze-cracking of some of the samples. Even though freeze-cracked samples were discarded, it cannot be completely ruled out that some samples had small internal cracks.

In an earlier paper, we depicted how the relaxation of fresh pomelo peel samples can be represented by a Maxwell model consisting of one hookean spring and three Maxwell elements [18]. Even though this is a phenomenological model, and thus the individual springs and dashpots cannot directly be related to the composition and the structural features of the plant tissue [19], it is useful for the interpretation of mechanical data. In a first order approximation, the springs can be assigned to the cell wall cellulose fibrils and microfibrils and the dashpots to the cell-wall matrix [25] and the cell sap contained in the protoplast. The finding that the COR of the impactor generally is higher for freeze-dried peel samples compared with fresh samples, which is an indication for a more elastic impact, allows to hypothesize that freeze-drying diminishes the viscous component of the pomelo peel. This is in line with the finding of Telis *et al.* [26] that dried foods generally are more rigid than fresh products and that they become more elastic, which was also shown for raisins [27,28] and prunes [29]. This shift in properties can be explained by the fact that forcing cell fluids through cell membranes and the cell walls is responsible for a large amount of the viscoelastic behaviour that plant cells exhibit when being compressed [17,30]. As stated by Niklas [23], evacuation of cell fluids through intercellular spaces, which in the case of pomelo peel are quite large, can also result in considerable viscous resistance. Figuratively speaking, freeze-drying removes the viscous liquid from the dashpots.

As the mesoscopic structural features of the peel samples remain in first approximation unaltered during freeze-drying [31], it can be said that the decrease of the COR with increasing impact velocity, is mainly based on these structural features and thus affects both fresh samples and freeze-dried samples. This overall loss in elasticity with increasing impact velocity can thus be regarded as plastic and/or visco-plastic failure of the sample. In fresh samples the cells probably burst under fast loading with high impacting forces, because the cell fluids have not enough time to evacuate from the protoplast as the permeability of the plasma membrane is limited [23]. The freeze-dried cells fail in a more brittle manner. The effect of moisture on artificial plant cell walls was shown by Cybulska *et al.* [32], who reported a marked increase in brittleness of artificial cell walls with decreasing relative humidity. The double peak in the force–time curve (figure 6) that resulted from compacting freeze-dried samples suggests shear failure within the sample. Plastic shear failure of freeze-dried samples extends the impact duration by creating macroscopic (internal) crack surfaces and thus leads to a reduced force peak especially for higher impact energies. Fresh peel samples tend to fail in a more uniform manner. These findings could be used for deriving a more sophisticated phenomenological model by incorporating friction, fracture and/or contact elements [33–35] or by introducing two-dimensional arrays of conventional elastic and viscous elements [17,35].

The finding that fresh peel samples were further compressed during impact than freeze-dried samples—at least for impactor drop heights between 0.125 m and 0.875 m where the difference is significant—results in an increased stiffness of the hookean springs of the Maxwell model for freeze-dried samples. It can safely be said that this is caused by the dehydration of the cell walls on a microscopic level, which is corroborated by Niklas [23] who stated that dehydrated tissues typically have higher Young's moduli. Also Lewicki *et al.* [36] found that water loss during drying resulted in rigidification of cell walls, which is probably due to the fact that only a highly hydrated, amorphous cell wall matrix allows for gliding or ‘slipping’ of the cellulose microfibrils. When the matrix of the cell wall is hydrated, the pectins of the matrix can behave as gel [23]. As a result, freeze-dried pomelo peel samples dissipate less of the kinetic energy of the striking impactor than do fresh samples.

The main task of every impact absorber serving, for example, as padding in the packaging industry or as personal protection gear that aims to reduce severe injuries is to keep the forces that occur during a drop or a crash at an undercritical level [37,38]. For an impact of a given energy, this means that it is favourable to extend the impact duration during which the force acts and thus to keep the force at every moment of the impact below the critical value.

Verification of the force sensor data by comparing *I*_i_ and *I*_mb_ (figure 7) shows that it is quite reliable and that the parameters extracted from the force curves can be trusted. It can be seen that the impact duration for fresh peel samples being compacted is strongly related to the sample height, while this is not the case for freeze-dried samples (or only to a very much lesser extent). This finding may be explained by a more uniform collapse of the fresh peel tissue that occurs during impact, i.e. these samples are uniformly deformed over their entire height as opposed to progressively collapsing freeze-dried samples where layer after layer is collapsing (cf. [39]). This behaviour is also reflected by the peak impact force that is decreasing with sample height in the case of fresh samples, whereas it stays more or less constant in the case of freeze-dried samples, probably also due to the aforementioned shear failure that occurs at a specific critical stress. The more fresh samples are compacted, the stiffer they become as cells in the whole sample are tautened [9], whereas during progressive collapsing in freeze-dried samples the failure of each layer happens at the same stress (owing to the anisotropy of the shrinkage, calculation of exact stress values would lead to spurious results). As the peel, however, exhibits a density gradient, the compacting pattern also depends on the exact layer of the peel that is being compacted; the less dense peel regions are collapsing first as the force acts on fewer struts and thus the stress for the individual strut is higher [18]. The comparably high peaks of force that occur during the impact of fresh samples when the impactor falls from relatively great heights is caused by the fact that the samples are bottoming out owing to their relatively low stiffness.

Finally, it should be pointed out that these results cannot directly be related to the effects that occur during the impact of a whole pomelo fruit: partly because also the fruit pulp deforms and is involved in energy dissipation, but mostly because the peel as such must be regarded as an integral functional unit. Excising samples from the peel in order to test them does not reflect the intact peel's mechanical behaviour in all its sophisticatedness but helps to understand the importance of specific peel structures and the dependence on cell and tissue properties as such, e.g. water content.

## 5. Conclusion

By performing drop weight experiments, it was shown that the water contained in the living cells constituting the peel parenchyma of the pomelo (*C. maxima*) peel has a strong impact on its mechanical properties. Based on the COR, it was found that freeze-drying clearly reduces the relative energy dissipation capacity of the peel. Measuring the transmitted force during impact furthermore indicated a transition from uniform collapse of the fresh foam-like tissue to progressive collapse due to water extraction in freeze-dried samples. Representing the peel as a Maxwell model led to the conclusion that freeze-drying not only drastically reduces the damping function of the dashpots but also stiffens the springs of the model. These effects are caused on the one hand by the lack of damping, which in fresh samples is caused by forcing cell fluids to flow through cell membranes and walls, and on the other hand, by changes of the mechanical properties of the cell walls.

## Data accessibility

The raw data supporting this article can be viewed at doi:10.6094/UNIFR/10090.
